# An Updated Review on the Role of Nanoformulated Phytochemicals in Colorectal Cancer

**DOI:** 10.3390/medicina59040685

**Published:** 2023-03-30

**Authors:** Alakesh Das, Suman Adhikari, Dikshita Deka, Nabajyoti Baildya, Padmavati Sahare, Antara Banerjee, Sujay Paul, Atil Bisgin, Surajit Pathak

**Affiliations:** 1 Department of Medical Biotechnology, Faculty of Allied Health Sciences, Chettinad Academy of Research and Education (CARE), Chettinad Hospital and Research Institute (CHRI), Chennai 603103, India; dasalakesh72@gmail.com (A.D.); dekadikshita14@gmail.com (D.D.); antara.banerjee27@gmail.com (A.B.); 2 Department of Chemistry, Govt. Degree College, Dharmanagar 799253, India; sumanadhi@gmail.com; 3 Department of Chemistry, Milki High School, Malda 732209, India; nabajyotibaildya@gmail.com; 4 Instituto de Neurobiología, Universidad Nacional Autónoma de México, Campus UNAM 3001, Juriquilla 76230, Querétaro, Mexico; padma.sahare@gmail.com; 5 Tecnologico de Monterrey, School of Engineering and Sciences, Campus Queretaro, San Pablo 76130, Querétaro, Mexico; 6 Cukurova University AGENTEM (Adana Genetic Diseases Diagnosis and Treatment Center), Medical Genetics Department of Medical Faculty, Cukurova University, Adana 01330, Turkey; abisgin@yahoo.com; 7 InfoGenom RD Laboratories of Cukurova Technopolis, Adana 01330, Turkey

**Keywords:** colorectal cancer, stress, chemotherapy, phytochemicals, nanotechnology

## Abstract

The most common cancer-related cause of death worldwide is colorectal cancer. It is initiated with the formation of polyps, which further cause the development of colorectal cancer in multistep phases. Colorectal cancer mortality is high despite recent treatment breakthroughs and a greater understanding of its pathophysiology. Stress is one of the major causes of triggering different cellular signalling cascades inside the body and which might turn toward the development of cancer. Naturally occurring plant compounds or phytochemicals are being studied for medical purposes. Phytochemicals’ benefits are being analyzed for inflammatory illnesses, liver failure, metabolic disorders, neurodegenerative disorders, and nephropathies. Cancer treatment with fewer side effects and better outcomes has been achieved by combining phytochemicals with chemotherapy. Resveratrol, curcumin, and epigallocatechin-3-gallate have been studied for their chemotherapeutic and chemopreventive potentiality, but hydrophobicity, solubility, poor bioavailability, and target selectivity limit the clinical uses of these compounds. The therapeutic potential is maximized by utilizing nanocarriers such as liposomes, micelles, nanoemulsions, and nanoparticles to increase phytochemical bioavailability and target specificity. This updated literature review discusses the clinical limitations, increased sensitivity, chemopreventive and chemotherapeutic effects, and the clinical limitations of the phytochemicals.

## 1. Introduction

Colorectal cancer (CRC) accounted for 10% of worldwide cancer incidence and 9.4% of cancer deaths in 2020 [1,2]. In terms of epidemiology, the CRC rate has increased over time, and in 2020, it was found to be more than 1.9 million, and it is considered to be the most commonly found cancer-related death, with what is estimated to be around 935,000 deaths [3,4]. In accordance with the developmental process of CRC, the overall rise in new cases is projected to increase to 3.2 million in 2040. The exposure to environmental risk factors brought on by a shift in lifestyle is primarily responsible for the rise in the incidence of CRC [1,2].

**Figure 1:**
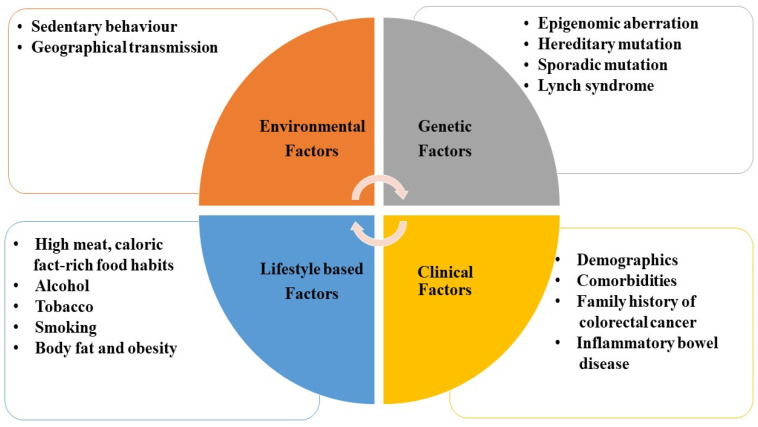
Schematic diagram representing a few of the risk factors associated with CRC.

Reports have suggested that CRC occurrence and mortality rates differ according to the geographic variation in locations around the globe, age, gender, and racial groups [5,6]. It was found that there is a significant enhancement in CRC incidence and mortality in countries with high and medium Human development index (HDI) that are more fascinated with the “Western” lifestyle [5,6,7]. In the modern world, economic growth followed by industrialization has led to the adaptation of Western dietary and lifestyle patterns, leading to a rise in the incidence rate of CRC in developed countries [3,7]. According to several reports, India, followed by Poland, in Asia or Europe, has a significantly enhanced CRC incidence rate with medium HDI. Countries such as the United Kingdom, India, Sweden, Germany, Australia, and a few others have a high incidence rate in younger people less than 50 years of age [4,6,7]. According to recent advancements, it is clear that the survival rate of CRC is based on the stage of diagnosis. Patients diagnosed at the early stages have a higher chance of survival than those diagnosed late [4,8]. Although there is an increase in the incidence rate, it does not necessarily translate to enhanced mortality rates in many countries, such as Slovenia and Italy, which might be because of improved CRC screening, leading to the detection of CRC at early stages. At the same time, countries such as Russia and Brazil have increased incidence and mortality rates with minimal medical sources [3,4,8,9]. Therefore, it can be concluded that proper medical and early screening facilities can help in reducing the mortality rates and the severity of the disease in almost all countries around the globe. According to reports, some of the well-recognized risk factors associated with CRC are environmental, dietary, genetic, and clinical factors, which are shown in Figure 1.

There has been much research on colorectal carcinogenesis since it can now be identified with the multi-step molecular mechanism of CRC development, including oncogenes and tumor suppressor genes. CRC has been associated with four critical genomic and epigenomic instabilities, including instability of the chromosome, CpG island methylator phenotype, microsatellite instability, along with DNA global hypomethylation [2,10]. Based on gene expression analyses, several CRC subtypes have also been found. Further, mounting evidence has also documented that increased reactive oxygen species (ROS) production is associated with CRC (references). Oxidative Stress (OS) is the result of ROS generation that surpasses the restricting capability of the antioxidant system of cells [10,11]. Generally, ROS in the intestine have been reported to have bactericidal effects that play a very significant part in the defense mechanism of the intestine, but OS generated from an overproduction of ROS surpasses the defense capability of the antioxidants, which leads to damage to the intestinal mucosal barrier and lipid peroxidation, followed by an inflammatory response and bacterial translocation [12,13]. An antioxidant defense mechanism is evolved in the cells for maintaining normal homeostasis among the antioxidant and oxidant species in the host [14,15]. The antioxidative and reactive oxygen species (ROS) scavenging properties of many phytochemicals such as curcumin, berberine, and tea polyphenols show chemo-preventive benefits in CRC. However, several different phytochemicals have been shown to promote ROS production and thereby trigger apoptosis in a variety of cell types. According to many studies, it has been concluded that ROS-generated genomic instability and DNA damage are associated with the initiation and progression of various cancers, along with CRC [16,17]. Early diagnosis of CRC is associated with fecal occult blood tests, sigmoidoscopy, and colonoscopy. Surgery along with pre or post-operative radio and chemotherapies are the main treatment methods available for CRC, but drug, chemo, and radiotherapy resistance are one of the major concerns for the existing therapies [18,19,20].

Hence, drugs coated with nanocarriers have attracted much interest in chemotherapy since nanoparticles have the potential for precise targeting, and lack drug resistance. Nanoscale compositions of drugs have lowered toxicity and improved bioavailability. Following the development of nanotechnology, some nanomedicines have been commercialized and some have entered clinical trials [21,22,23]. By enabling drug combination therapy and inhibiting drug resistance mechanisms, nanomedicines have advanced drug delivery methods and multidrug resistance (MDR) factors [24]. Despite the fact that a large number of studies indicate that nanomedicine therapies are beneficial in the treatment of cancer, both in vitro and in vivo, relatively few nanocarrier-based cancer treatments have entered clinical trials. The most widely recognized drawback of nanomedicine is its potential to interact with several other biomolecules or biological fluids, resulting in particle aggregation or agglomeration. Such interactions have the potential to dramatically affect the function of nanomedicine molecules in biological systems [24,25]. As a result, they are critical to solving the issues associated with developing optimal nanomedicine products for clinical application.

Phytochemicals, which can be found in foods, including vegetables, fruits, cereals, and other parts of plants, have been shown to have anticancer effects on several kinds of cancer [26]. Several studies have shown that these phytochemicals can control various molecular and cellular processes which include the proliferation of cells, repairing of DNA, apoptosis, and metastasis [27]. Moreover, they are typically beneficial to normal physiological activities, and when used in required dosages, they have minimal or no toxicity in humans. Further study into the pharmacological mechanisms of natural anti-tumor activities of phytochemicals has documented their ability to target several cancer-related signaling pathways and play antioxidation, anti-inflammation, and immune-modulatory roles. Despite the significant therapeutic promise, various difficulties, including low absorption and cell penetration, limited solubility in water, and their chemical instability must be solved before their application as anti-cancer therapy as a supportive drug [27,28,29].

Numerous studies have shown that phytochemicals that are loaded in nanoformulation are found to enhance pharmacokinetics and biodistribution with solubility, contributing to their high therapeutic index. The field of phytochemical-loaded nanoformulations, often known as phytonanomedicine, has recently received much consideration. Additionally, phytonanomedicine demonstrates a decrease in toxicity and an increase in phytochemical efficacy [21,23,30]. Therefore, the nanoformulation of phytochemicals might overcome their disadvantages and can be a possible antitumor treatment candidate taking the benefit of nanoparticle-based delivery of drugs into account [31]. Therefore, in this review, we focused on discussing the potential of nanoformulated phytochemicals as novel agents for a supportive therapy for CRC. In addition, it also discusses the limitations, increased chemosensitivity, and advantages of nanoformulated phytochemicals and their role in CRC.

## 2. Limitations of the Existing Therapeutic Modalities for CRC

Depending on the pathological features of the tumor, there are numerous therapy approaches for CRC. Laparoscopic surgery is often used for early-stage primary illness, open surgery for metastases, and adjuvant radiotherapy for nonresectable patients. In CRC, acquired drug resistance is a common problem. Advanced CRC is often treated with fluoropyrimidines (5-FU or capecitabine) in conjunction with various other drugs such as oxaliplatin, topoisomerase I inhibitor irinotecan, monoclonal antibodies CTX, BEV, or panitumumab, along with a few newer drugs that are considered for administration such as aflibercept and regorafenib [18,23,32]. Even though these newly produced biologics have enhanced the patients’ prognosis, resistance to drugs is still a substantial problem in treating CRC patients. Initial responders with advanced stages of CRC revealed that the disease progresses within a short period of time [33]. The emergence of drug-resistant phenotypic and genetic mutations in cancerous cells is thought to be the major cause of the progression of cancer. Patients who were treated with anti-EGFR mAb had genetic markers of acquired resistance in their tumors. Even if they had a sensitive primary tumor, they could only show a measurable response for 3–12 months before the progression of the tumor. Several molecular mechanisms cause chemoresistance in tumor cells, comprising drug efflux and influx regulation via cell death suppression, the ATP-binding cassette transporter family, epigenetic factors, changes in drug absorption and metabolism, reduced functionality of the chemotherapeutic agents, mutations in the target of the drug, increased DNA repair, and shifts in growth factor signaling. The mentioned aspects can perform unilaterally or in conjugation via diverse signaling cascades. Chemoresistance is a key constraint of chemotherapeutic treatment modalities, compelling scientists to emphasize developing a safe and novel product that can repress chemoresistance and significantly sensitize tumor cells to chemotherapy regimens. Chemosensitization is an extensively utilized approach to boost the function of one drug by integrating it with another drug in order to surmount chemoresistance [32,33,34]. Although several studies have provided evidence of acquired drug resistance in CRC, further in-depth analysis is still required to pave the way for novel approaches to reducing the recurrence rate of CRC and increasing the overall survival rate. Therefore, to overcome multidrug resistance or chemoresistance it is crucial to evaluate an alternative approach, such as nanoformulated drugs or rather nanoformulated phytochemicals, which may bypass the chemoresistance mechanism and can act through a new strategy to target the cancer cells with lesser toxicity and enhanced bioavailability [1,2,10,18,19,20,34].

Recent research has highlighted the fabrication and function of several phytochemical compositions that have evolved by combining precise antibodies, adjuvants, or even other natural compounds in a single molecule for improving bioavailability and solubility and, in certain instances, to decrease cancer cell resistance by offering a chemosensitizing effect. These novel regimens have the possibility of enhancing the pharmacodynamics of phytochemicals for interventional purposes in the future by overcoming the limitations of pharmacokinetics [1,2,10,18,19,35,36,37]. For example, curcumin bioconjugated with luteinizing hormone-releasing hormone (LHRH) analog [DLys6]-LHRH impeded growth and the induction of apoptosis in pancreatic cancer cell xenografts. The efficiency of curcumin-[DLys6]-LHRH bioconjugate was observed to be similar to free curcumin, and this combination also displayed enhanced solubility. The addition of folic acid to curcumin entrapped in polyethylene glycol-polylactic acid-co-glycolic acid nanoparticles considerably improved curcumin’s efficiency in repressing numerous survival impulses stimulated by PTX in cervical cancer cells and substantially increased its capacity to decrease tumor progression in cervical cancer xenografts [38,39,40,41,42]. A nanoparticle formulation based on curcumin and lipoic acid crosslinked to human serum albumin has been demonstrated to intensify the in vitro antitumor effects of Doxorubicin against H295R adrenocortical tumor cells and demonstrated the effective and fast uptake of the nanoparticle. Furthermore, hybrid nanoformulated phytochemicals are known to facilitate the reversal of chemoresistance to different drugs in various types of cancer. As a result, it is a potential approach for combating treatment difficulties correlated with the therapeutic applications of nanoformulated phytochemicals [38,39,40,41,42,43,44,45,46,47,48].

## 3. Significance of Nanomaterials in Colorectal Cancer Research

Nanotechnology has revolutionized several scientific achievements, the most noteworthy being the invention of nanomedicine. Specifically, nano-drug delivery for cancer treatment has been investigated. Nonetheless, more study is necessary to improve the interaction of phytochemicals with cancerous tissues though minimizing their adverse effects on healthy organs. Because of the pathological and physiological properties of the tumor, passive targeting using the enhanced permeability and retention (EPR) effect is often used for phytochemical release [49]. Additionally, active targeting may be accomplished by incorporating compounds that attach to overexpressed cancer cell surface receptors. Active targeting may further increase the phytochemical content at the site of the tumor. Enhanced cellular internalization may substantially improve phytochemical cell exposure and bioavailability. CRC has been treated using liposomes administered orally, intravenously, and rectally. Panitumumab and cetuximab (Cxm) are monoclonal antibodies that target ERBB1 (EGF receptor) signalling, which is essential to the development and progression of CRC [1,50]. A study examined the anticancer effect of triptolide and triptolide-loaded polymeric micelles in human HT29 adenocarcinoma cells [51]. Using HCT116 human colon cancer cells, the authors reported the antiproliferative properties of the bioactive phytochemicals thymoquinone integrated into poly lactide-co-glycolide (PLGA) nanoparticles. The encapsulation efficiency of TQ-PLGA nanoparticles ranged between 94 and 95 percent, and their diameters ranged from 150 to 200 nm. The nanoparticle TQ-PLGA displayed anticancer properties in HCT116 cells. TQ-PLGA was much more effective than free TQ in hindering NF-κB activation and decreasing the production of matrix metalloproteinase (MMP-9), cyclin D1, and vascular endothelial growth factor (VEGF) [52,53].

## 4. Nanomaterials: Types and Its Applications in CRC

**Figure 2:**
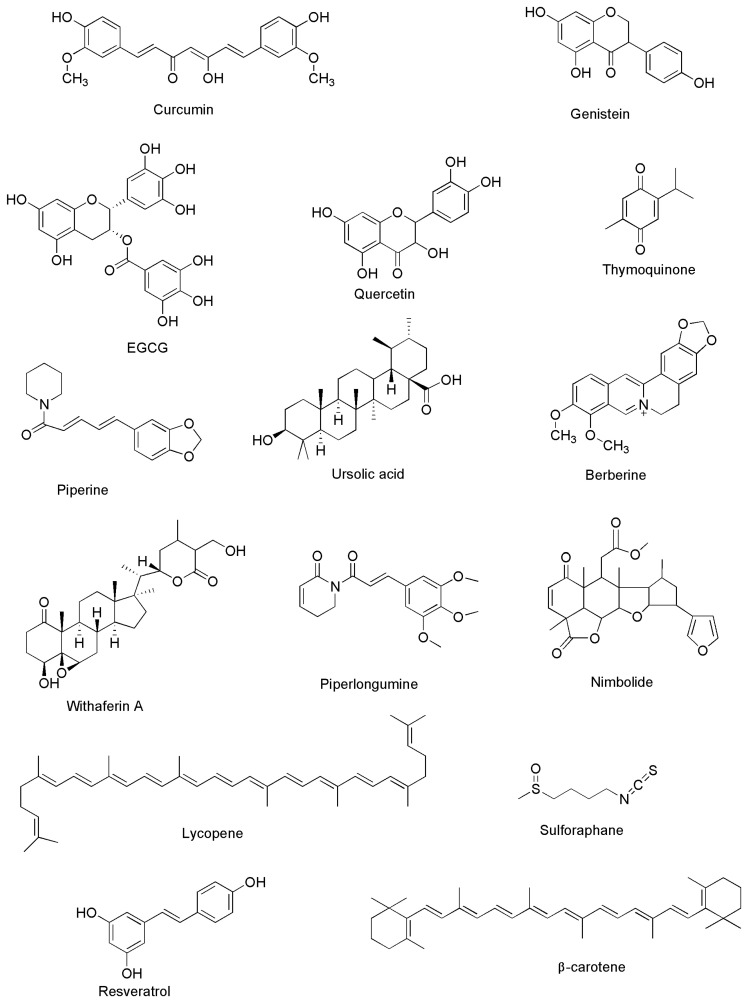
Diagram representing the chemical structure of a few of the well-documented phytochemicals used for CRC treatment.

Nanomaterials have gained global interest owing to their capacity to improve conventional CRC treatment approaches. Some of the most intriguing nanomaterial-based therapeutic techniques include photothermal treatment, medication, and gene delivery system magnetic hyperthermia, and photodynamic therapy [10,54,55]. The development of tailored targeted drug delivery systems and cutting-edge treatment methods are some of the larger uses of nanomaterials in the treatment of CRC, as shown in Figure 2.

Some nanostructures are better suited for specific drug delivery or tumor-screening applications than others, such as carbon nanotubes, liposomes, polymeric nanoparticles, metal–organic frameworks, silica nanoparticles, lipid nanoparticles, lipid bilayer, dendrimers, quantum dots, and nanocapsule, among which a few are discussed below. In addition, all of these targeted drug delivery systems have opened the way for innovative nanoformulations, which have recently emerged as the focus of scientific investigations.

### 4.1. Liposomes

Traditional liposomes are phospholipid, amphiphilic, and spherical vesicles with a 25–2500 nm diameter that forms a closed bilayer around hydrophobic or hydrophilic molecules to protect them from aqueous or non-aqueous surroundings, respectively. The number of lipid bilayers in these closed vesicles determines whether their structure is either multilamellar or unilamellar. Unilamellar systems with an aqueous core include water-soluble drugs; on the other hand, multilamellar systems contain drugs that are lipid-soluble. After surface coating with protein and intravenous administration, liposomes are immediately removed by the reticuloendothelial system (RES). Liposomes can also be disassembled by hydrophobic, electrostatic, and van der Waals forces. Thus, steric stabilization is necessary, which can be accomplished by covering the particles with inert polymers [56,57]. Liposomes have been proven effective and safe as a biocompatible and biodegradable drug delivery system without the risk of immunogenicity or toxicity. Pharmaceuticals and imaging agents, function as a framework, extending their half-life and allowing for more precise targeting. The lipid-based methodology has now been used to evaluate various cancer therapies utilizing different preparation procedures. Examples include the successful use of liposomal daunorubicin and doxorubicin for metastatic breast cancer as well as AIDS-associated Kaposi’s sarcoma. Several other liposomal chemotherapeutics are now being studied in clinical trials, and these are already-approved medications [58,59,60,61]. Studies on the use of nanomaterials in CRC have shown the potentiality of a liposomal nanocarrier in reducing COX-2 and HIF-1 in CRC cells. A decrease in tumor growth was also found in the mouse model when treated with dual drug-loaded liposomes compared to single drug-loaded liposomes. Moreover, combining liposome-formulated drugs with radiotherapy was found to show a better effect than stand-alone for CRC [56,57,62,63]. Further, immunoliposomes that only release the medicine to the targeted action sites could represent the next generation of liposomal medications.

### 4.2. Micelles

Micelles are self-aggregating amphiphilic block copolymers that self-assemble into core/shell structures at the nanoscale, with typical diameters ranging from 10 to 100 nm. The core is a dense hydrophobic region, whereas the shell consists of hydrophilic copolymers. Adding different chemical linkages, such as esters, to the surface of polymeric micelle liposomes, makes it possible to modify blood stability and drug release rate. The three copolymers that have been the subject of most investigation are poly(esters), poly(L-amino acids), and poly(propylene oxide). The hydrophilic shell of the polymeric micelle (PM) and its nanoscopic size inhibit the mechanical clearance of the micelles via reticuloendothelial system absorption, renal filtration, and the spleen, enhancing their circulation in the blood for a long period of time [64,65,66]. General PM, which is paclitaxel in the PM form, is an anti-cancer medication with a PM formulation. Paclitaxel, a chemotherapeutic agent with limited water solubility, is utilized to treat ovarian cancer, breast cancer, Kaposi’s sarcomas associated with AIDS, and non-small cell lung cancer [58,59,60,61,66,67]. A large number of agents having anticancer properties have poor solubility in an aqueous medium, whereas polymeric micelles are found to be powerful vehicles in the loading and delivery of drugs that are hydrophobic in nature. On the other hand, they are highly biocompatible, and biodegradable, and can be useful in the stimuli-responsive targeting of tumor moieties, gene delivery, and imaging. Genexol-PM loading paclitaxel (PTX) and Nanoxel loading docetaxel (DTX) are the only polymeric micelles approved for treating a number of cancers in India and Korea. They are found to have the potential to be used as therapeutics against solid tumors, and they can be used to deliver drugs overcoming the restrictions of CRC chemotherapy [68].

### 4.3. Dendrimers

Dendrimers are three-dimensional tree-like structures having multifunctional, regularly branching central core molecules. The branching units are thought to originate from the core molecule via polymerization or arise from the periphery and end at the core molecule [69,70]. The drug molecules can be joined to functional groups on the surface of the dendrimer or protected in the interior environment of the dendritic channels of the sphere. Like liposomes, dendrimers can enclose water-insoluble chemicals in their interior cavities or bind them to their surface via electrostatic or hydrophobic interactions. This increases the solubility and bioavailability of the compounds. Dendrimers can also transport nucleic acid-based chemotherapies that are having trouble passing through the cell membrane [71,72,73,74,75]. Further, a number of studies have suggested that dendrimers will be a novel candidate for treating cancer in the future as dendrimer nanoparticle-based diagnostic and therapeutic strategies in the field of oncology [76]. One of the most common targeting strategies will be its association with the anti-metabolite drugs, hormones, or vitamins that are necessary for the growth of the tumor [77].

### 4.4. Nanospheres

The medication is disseminated through entrapment, attachment, or encapsulation in these nanoparticles‘ spherical structures made of a matrix system. Polymers can be added to the sphere’s surface, and biological components such as antibodies or ligands can be bound for targeting purposes [78,79]. Nanospheres have also been found to be useful in understanding their capability in the modulation of cancers, including CRC, in recent years. Among them, zinc oxide nanosphere and gold nanodot decorated hollow carbon nanosphere are the most recently analyzed against CRC [78,79,80].

### 4.5. Quantum Dots

Quantum dots (QDs) are nanocrystal-sized semiconducting materials possessing an organic-coated and inorganic core/shell. When light is used to excite QDs, it emits fluorescence. QDs’ one-of-a-kind property makes them a helpful tool for tracking and imaging intracellular mechanisms. QDs can aggregate in tumor tissue, thus allowing non-invasive tumor detection and visualization [79]. In vitro studies using QDs for fast localization of HER-2 receptors, imaging-guided therapy, and targeted chemotherapy have yielded promising results [81]. QDs are also documented to have been successfully used against a number of cancers. A study showed that graphene oxide quantum dots, when covalently labeled with a peptide, increased the cytotoxicity and reduced the invasion of HT29 and HCT116 cells. Carbon quantum dots have recently gained attention because of their potential in a number of fields, including cancer; still, in-depth analysis is required to unfold their other advantage to be used as therapeutics for CRC management [82].

## 5. The Significance and Limitations of Phytochemicals in CRC Treatment

**Figure 3:**
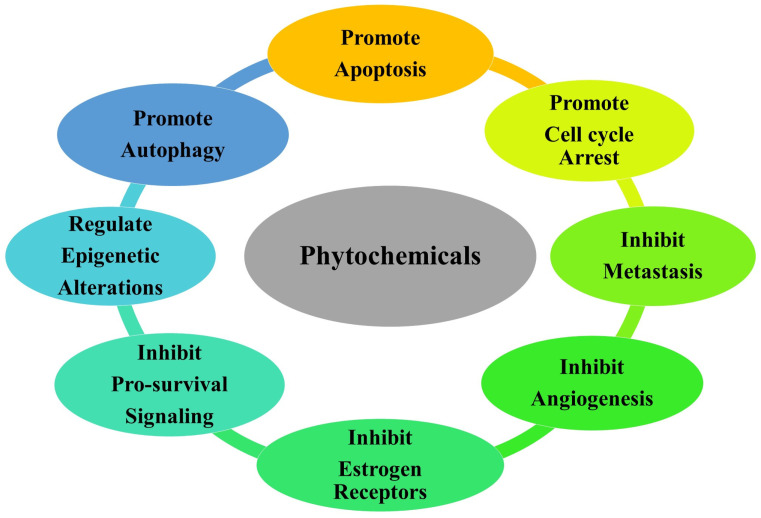
A schematic diagram representing the various functions of phytochemicals involved in the regulation of molecular pathways associated with cancer.

Initially, identification of the bioactive phytochemicals is made before being utilized as a basis for modification of the structure or as lead chemicals used for the syncretization of molecules based on physicochemical or structure–activity relationships, in addition to pharmacodynamics features to reduce the toxicity, as well as enhance the bioavailability of the identified bioactive phytochemicals [83]. Studies have documented that several phytochemicals possess chemosensitizing capability in several cancers, such as CRC, liver cancer, breast cancer, bladder cancer, and many more, as shown in Figure 3. Among the phytochemicals, curcumin is widely studied for its chemosensitizing capability and anti-inflammatory, antioxidant, anti-angiogenesis, and antiproliferative properties [22,25,84]. Along with it, several other phytochemicals such as apigenin, berberine, betulinic acid, resveratrol, tea polyphenols, celastrol, quercetin, and many others have been found to have potential to be used as an anti-cancer candidate for drugs in combinational treatment in several cancers [85]. It has been found that the major problem in enhancing the treatment efficacy of CRC is chemoresistance to the drugs. Signalling pathways such as mitogen-activated protein kinase kinase kinase 8 (MAP3K8), NF-κB, and Akt are linked to the development of the disease and resistance to a number of medications used for CRC treatment [86]. Phytochemical apigenin is found to inhibit the Akt/ERK signalling pathway in both in vitro as well as in vivo analysis [87]. Betulinic acid was found to reverse chemoresistance in colon cancer cells [88]. Together, boswellic acid and curcumin have the capability to act as an anti-tumorigenic agent and modulate cancer miR-27a and miR-34a that are associated with cancer [89,90]. Curcumin and EGCG have been reported to induce chemosensitivity in both combinations and as a stand-alone treatment of CRC. Genistein is also found to have a number of beneficial effects on several diseases, including colon cancer. It also possesses the capability to inhibit NF-κB resulting in the initiation of apoptosis in human colon cancerous cells and T-cell leukemia cells. Another phytochemical, lycopene, has been reported to have anti-proliferative and anti-tumor properties [85,91,92].

An epidemiological study demonstrates a significant correlation between a fruit and vegetable diet and a reduced threat of CRC. Moreover, multiple in vitro and in vivo analyses show that phytochemicals exert powerful antioxidant and anti-inflammatory effects by regulating key signalling pathways to prevent the genesis and progression of CRC. Numerous studies have concluded that, in the future, chemopreventive research should focus on creating innovative strategies that target the molecular pathways connected with cancer, as well as implementing nutritional therapies for large populations afflicted with CRC [93,94,95]. Diverse phytochemical compositions are now being investigated for potential application in cancer treatment, but several issues must be resolved first. These challenges include a restricted therapeutic index, low solubility, poor cell penetration, hepatic disposition, and fast absorption by normal tissues. Due to the substantial drug aggregation in healthy organs, phytochemicals have a large apparent volume of distribution. Phytochemicals may have poor pharmacokinetic features as a small drug molecule, which may have a detrimental impact on their therapeutic index [96,97,98]. Vincristine, for example, is utilized for solid tumor treatment as well as hematologic malignancies. Still, its use as an anti-cancer medicine has been limited by its unfavorable pharmacokinetic features and dose-related neurotoxicity. It has restricted solubility in aqueous solutions at physiological pH and is based on its pharmacokinetics and a very short distribution of half-life and a comparatively longer elimination. A recent study has shown that using phytochemicals in clinical practice is hindered by multidrug resistance [99,100,101]. However, with time, cancer cells may resist certain anti-cancer medications, resulting in treatment failure. This is mainly attributable to rapid drug metabolism, changed molecular targets, over-expressed efflux pumps, gene deletion, genetic anomalies, and decreased apoptosis. Alternatively, tumor malignancies can be caused by cell–cell contact, low pH and high pressure in interstitial fluid, and uneven tumor vasculature, among other reasons. Therefore, an excellent phytochemical formulation should possess the capability of overcoming these barriers for maximum clinical anti-carcinogenic efficacy and minimal side effects [85,102,103].

## 6. Need of Phytochemical-Based Nanoformulations for CRC Therapy

Bioactive compounds derived from plants, such as vitamins, flavonoids, minerals, terpins, alkaloids, gums, oils, glycosides, lignins, saponin, podophyllotoxin, taxol, vinca alkaloids, and polyphenols, play the function of inhibition as primary and secondary metabolites. Phytochemicals actively inhibit or overexpress certain kinds of enzymes, proteins, or other metabolites in the CRC cell. Phytochemicals have no harmful effects on normal cells. The main mechanism of phytochemicals may be triggered in a variety of ways [104]. Natural phytochemical qualities, their impact on the body, and research on their passage into the body are critical. Poor oral bioavailability and inadequate intestinal absorption have hampered the development of natural compounds to date [105]. Therefore, nanoformulated-based phytochemicals have recently become a major concern for their effective role in regulating or treatment of CRC.

Researchers often refer to the use of nanotechnology in health care as nanomedicine. The term nanoparticles (NPs) refers to particles with one dimension smaller than 100 nm and having special characteristics that are typically absent from bulk compounds of the same material [106]. NPs have grown in significance in an extensive range of research arenas due to their distinctive qualities, including high surface-to-volume ratios, sub-micron sizes, and enhanced targeting systems. By adjusting the composition, particle shape, size, and surface properties of the NPs, it is possible to maximize their performance, improve therapeutic effectiveness, lessen adverse effects, and overcome medication resistance. Because natural substances made at the nanoscale exhibit radically changed bioactivities and toxicity, the development of a nanoparticle-based therapeutic transport system holds the potential for cancer treatment in this scenario. Drugs can circulate in the body for a more extended period of time thanks to NPs, which also shield them from external physiological harm. Additionally, NPs have the benefit of delivering medications that are not water soluble, assisting them in crossing biological membranes, enabling the distribution of one or more drugs simultaneously, and achieving target-precise drug release [106,107]. Research on nanotechnology has demonstrated the enormous advantages of nanoparticle-based delivery methods over traditional therapies. Drugs enclosed in nanoparticles can be shielded from the damaging effects of outside media, which is one benefit of this nanotechnology. As a result, the drug’s half-life systemic circulation can be extended. Additionally, it is now well recognized that nanoparticles can enhance water-insoluble drug delivery, supply continuous release of a drug, improve the passage of chemotherapeutic medications across cell membranes, improve the distribution of drugs, facilitate the drugs to be delivered only to the cancerous cells, and assist in two or more drug deliveries for combined therapy as opposed to non-encapsulated free drugs [108,109,110].

**Figure 4:**
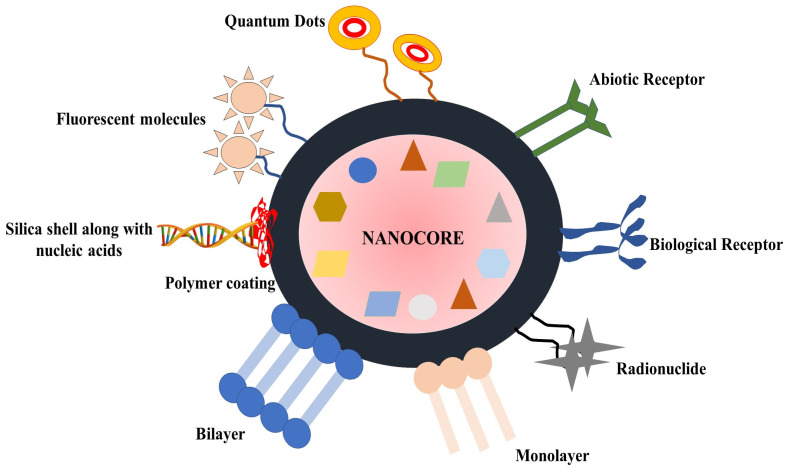
Schematic diagram representing multifunctional nanoparticle.

It has been demonstrated that nanotechnology makes the development of cancer drugs more successful. Compared to free phytochemicals, phytochemical nanoparticles exhibit improved solubility in addition to other benefits. The formation of nanoparticles alters the pharmacokinetics and biodistribution of phytochemicals, increasing their therapeutic index by reducing toxicity and raising efficacy, as shown in Figure 4. For instance, creating antibody–drug conjugates (ADCs) by combining phytochemicals and antibodies can deliver powerful cytotoxic phytochemicals to cancerous cells in a targeted manner. The diameters of these conjugates are in the nanometer range. High-potency cytotoxic phytochemicals have a wider treatment window because ADCs are more selective for tumors than other organs [63,85,111]. The main method used by nanovehicles to tumor targets is the superior permeability and retention (EPR) effect. Most solid tumors have insufficient lymphatic drainage and blood vessel architectural defects. The nanocarriers and macromolecules can leak preferentially from the blood arteries neighboring a tumor [55]. Meanwhile, tumors’ inadequate lymphatic drainage enables them to congregate close to the cancer cells. Normal tissues do not experience an EPR effect. The EPR effect is primarily influenced by two factors: size and biocompatibility. It is necessary to have an extended circulation period in order to provide sufficient time for the EPR effect to distribute the drug. The majority of phytochemicals have low molecular weights, have a rapid clearance rate in living organisms, and are widely distributed throughout healthy tissues and organs [112]. Here, we have discussed a few of the extensively studied nanoformulated phytochemicals against CRC.

### 6.1. Curcumin

Curcumin has been discovered to have chemopreventive and anti-cancer qualities, whether used alone or in combination, and has also been used for the treatment of cancers such as pancreatic, breast, colorectal, lung, and prostate cancer [113,114,115,116,117]. Curcumin inhibits various cell signalling pathways, including STAT3, NF-κB, epidermal growth response-1, and activated protein-1, which are essential for the genesis and development of tumors. These transcription features are known to be elevated in most malignancies to promote cell proliferation, angiogenesis, and tumor development [38]. Curcumin exerts its antioxidant properties via diffusing cells into the mitochondria, endoplasmic reticulum, and nucleus. It has therefore been advocated as a chemopreventive, antimetastatic, and antiangiogenic drug [39]. Curcumin inhibited cellular development by arresting the cell cycle in the G2/M and G1 phases and triggering apoptosis by interacting with many molecular targets. In vivo studies demonstrating a chemopreventive impact on genetic and inflammatory CRC animal models have already been undertaken. Curcumin has also been conjugated with tiny particles to increase its oral bioavailability, significantly affecting inflammation and carcinogenesis. It has been included in dietary formulations for CRC chemoprevention [38,39,40,117]. These combinations demonstrated anti-carcinogenic properties in in vitro and in vivo models of inflammation and hereditary-related CRC.

Liposomal formulations increased curcumin’s activity in in vitro studies against HCT116 and HCT15 cell lines. It was also proven that the integration efficiency of lyophilized liposomal curcumin production is around 85%. Furthermore, liposomes increased the cytotoxic activity of curcumin in colorectal cancerous cells, showing that it is more effective than free curcumin [41]. Similarly, curcumin and piperine were synthesized as emulsions to enhance their limited absorption and provide a synergistic anti-cancer effect in vitro colon cancer model. Piperine emulsomes augment the anti-cancer effects of curcumin on HCT116 cells when combined with CurcuEmulsomes [42]. Liposomes encapsulating curcumin and doxorubicin showed a more significant cytotoxic effect than doxorubicin-loaded liposomes at higher curcumin concentrations, as shown by the analysis of curcumin’s capacity. A microfluidic hydrodynamic approach for creating nano-sized liposomes that are dual-loaded with curcumin and catechin is described in research that shows a synergistic effect on colon cancer cells. In addition, HT29 and Caco-2 were used to test the anti-cancer effect regarding cytotoxicity in which it was found that, in a concentration-dependent method, the cytotoxic result of dual-loaded liposomes on HT29 and Caco-2 cells was greater in comparison to the two single compounds [43,44]. Curcumin nanoformulations are also designed to enhance therapeutic advantages while decreasing the risk of severe side effects by delivering curcumin to the target region. Due to the increased absorption and targeting of curcumin nanoformulations after oral administration, in vivo studies demonstrate its potential. Some investigations show that these nanoformulations might be employed to treat other chronic and life-threatening diseases [45]. Curcumin was co-encapsulated with piperine (PIP) in poly allylamine hydrochloride (PAH) nanocapsules and evaluated on the Caco-2 cell line, reducing vitality with increasing PIP concentration. In addition, PIP enhanced cytotoxicity while enhancing accumulation in colon cancer cells. As a result of the information presented here, curcumin or derivatives in nanoformulations may probably be the subject of future clinical trials in search of a safer and more effective curcumin therapy for CRC [46,47]. Various clinical trials have evaluated the efficacy of substances on CRC, as clinical studies bridge the gap between the laboratory and the market [48,118]. Therefore, extensive human clinical studies are required to demonstrate their safety, particularly after long-term and repeated usage, and their efficacy in treating colon cancer and other disorders.

### 6.2. Resveratrol

The activation of many signalling pathways is associated with the genesis and progression of various malignancies, including CRC. Therefore, blocking these signalling pathways using non-cytotoxic natural products is a practical method for preventing and treating colon cancer. Resveratrol, a polyphenol found in grapes, inhibits the proliferation of cells, triggers growth arrest, and induces apoptosis in a range of malignant cells [23,119]. The primitive study discovered that resveratrol has anti-cancer capabilities against several tumor forms and impacts many stages, from tumor start and proliferation to metastasis. Resveratrol, in particular, can induce the death of cancer cells by interfering with signalling pathways that are active in cancerous cells. It inhibits Wnt signalling in RKO cells and NCM460 cell lines lacking intrinsic Wnt pathway activity [119]. The activation of autophagy has also been shown in HT29 and COLO 201 cells; however, the amount of Ras-induced autophagy and apoptosis differs between colon cancer cell lines [120]. Another study showed that relatively high concentrations of resveratrol might, for the first time, considerably inhibit telomerase activity. These preliminary results imply that resveratrol may play a role in the treatment of human colon tumor cells and offer a basis for developing new methods for cancer management. Further, in vivo research indicated that the combination of resveratrol and DHM (1,2-dimethylhydrazine) slowed the growth of intraepithelial neoplasia in colon tissue, indicating the potential of resveratrol for CRC prevention [121]. To comprehend the molecular mechanism by which resveratrol slows the growth of colon cancer, further research is necessary.

Resveratrol stimulates the oxidation of fatty acid in SW620 colon cancer cells, researchers found in an intriguing study. It also showed that it might promote the generation of ROS and the mitochondrial membrane, cause apoptosis, and obstruct cell growth. These findings provide insights into how resveratrol fights cancer by showing that it may target cancer cell metabolism and boost chemotherapy effects [122]. Resveratrol was encapsulated in PLGA-polyethylene glycol (PEG) NPs coated with chitosan (NP-RSV) and was examined with human COLO205-Luc colon cancer in an orthotopic implantation and xenograft model in athymic mice to investigate the antiproliferative and anti-angiogenic properties of resveratrol and its nanoformulation. The outcomes showed that NP-RSV has significant anti-cancer and antiangiogenic properties [123]. Separate research found that liposomal nanoparticles improve the chemotherapeutic effects of resveratrol on colon cancer cells by using a dual encapsulation method. Additionally, the MCM-48 mesoporous silica nanoparticles dramatically enhanced resveratrol’s solubility. As RES dissolved in DMSO, MCM-48-RES was found to enhance dose-dependent cytotoxicity in cell lines. The increased apoptosis rate caused by MCM-48-RES was validated by immunoblotting proteins involved in the apoptosis pathway [124,125]. Resveratrol in its encapsulated form showed a more substantial anti-cancer impact on HT29 cancer cells than resveratrol in its free form. Colloidal mesoporous silica nanoparticles pre-loaded with resveratrol increased the substance’s solubility by a factor of two and delayed its release compared to pure resveratrol. In a study, resveratrol-loaded gold nanoparticles were characterized and tested for their ability to target HT29 cells in a mouse cancer model. Cellular absorption of the resveratrol gold nanoparticle was shown to be much greater than that of pure gold nanoparticles or resveratrol alone. Moreover, resveratrol gold nanoparticles delivered to rats with colon tumors produced more enhanced in vivo targeting than resveratrol 99 m technetium-tagged resveratrol. Mesoporous silica nanospheres loaded with resveratrol were created, and it was shown that the saturation solubility of the resveratrol in the nanospheres depended on pore size and particle size [126,127]. Additionally, resveratrol-loaded nanospheres improved the permeability of a monolayer of human colon cancer cells. Compared to the suspension and solution, resveratrol encapsulation exhibits a more substantial anti-inflammatory impact [128]. Resveratrol-loaded PEG-polylactic acid-based polymeric nanoparticles were developed to restrain glucose metabolism and the growth of a tumor, leading to an increase in apoptotic cell death [129,130]. However, more in vivo research is required to confirm the improved advantages of resveratrol nanoformulations.

### 6.3. Tea Polyphenols

Historically, people have turned to tea as a means of maintaining health and combating illness. Tea has been demonstrated to have several positive effects, including antioxidant, bacteriostatic, anti-cancer, and lipid metabolism regulatory effects. Tea polyphenols (TPs), also called catechins, are flavonoids with the structure of α-phenyl-benzopyran. They make up between 18% and 36% of the dry weight of tea leaves. The major tea catechins are epigallocatechin-3-gallate (EGCG), epigllocatechin (EG), epicatechin-3-gallate (ECG), and epicatechin (EC). Green tea has been discovered to have properties that deactivate enzymes and prevent the oxidation of tea elements [131,132]. TPs can prevent CRC from growing and spreading by modulating anti-oxidative or pro-oxidative, anti-inflammatory, and pro-apoptotic actions. Numerous studies have shown that TPs can control several signalling pathways, including the 67 kDa laminin receptor pathway, the phosphatidylinositol-3 kinase/Akt cascade, the Wnt/β-catenin cascade, the mitogen-activated protein kinase path, and the phosphatidylinositol-3 kinase/Akt pathway, to the inhibit proliferation of cells and promote cellular apoptosis. Additionally, a recent study suggests that TPs can inhibit CRC growth and metastasis by altering the composition of the gut microbiota, which strengthens the immune system and reduces inflammation [132,133,134]. Dietary polyphenols have been shown to affect epigenetics, inflammation, mRNA expression, and gut flora, all of which have been linked to anti-cancer properties [134,135,136]. Numerous innovative cancer prevention and treatment options have been made possible by the ongoing identification and analysis of polyphenols. Clinical anti-colorectal cancer investigations are increasingly using polyphenols and their derivatives. For instance, silymarin may suppress Wnt signalling in CRC cells, lowering the production of hydro-catenin and TCF4, and leading to tumor cell death. Epigallocatechin-3-gallate reduced the growth of Hs578T breast ductal carcinoma and Caco-2 colorectal adenocarcinoma cells. It is essential to investigate further if the TPs’ metabolites also have antiproliferative effects in addition to the TPs themselves [137,138,139].

Among the various catechins, EGCG has gained the most concern in the field of research due to its potential to act as an anti-cancer agent. It was discovered that using epigallocatechin-3-gallate in conjunction with radiation therapy, which also stimulated the nuclear translocation of Nrf2, and the expression of genes linked to autophagy, prevented the proliferation of CRC cells. Additionally, epigallocatechin-3-gallate boosted radiation sensitivity in CRC cells by decreasing cell survival, enhancing Nrf2 nuclear translocation, and upregulating genes involved in autophagy [140]. According to one study, epigallocatechin-3-gallate may prevent colorectal cancer cells from forming spheroid-like structures. Additionally, it has been suggested that epigallocatechin-3-gallate may be a potential drug to treat CRC since it works by blocking the Wnt/β-catenin pathway to target colorectal cancer stem cells (CSC) markers [141]. The synergistic impact of epigallocatechin-3-gallate with cisplatin and oxaliplatin is also a result of autophagy in DLD-1 and HT-26 cells. The cytotoxicity of cisplatin and oxaliplatin in CRC cells is subsequently increased by epigallocatechin-3-gallate-induced autophagy via an autophagy-related mechanism [142]. Another study found that green tea extract (GTE) consumption over a long period may help the Korean population avoid colorectal adenomas since it is well-tolerated and safe for CRC prevention [143,144]. According to research, chitosan nanoparticles encapsulating epigallocatechin-3-gallate (Chit-nanoEGCG) significantly increased poly (ADP-ribose) polymerases cleavage in tumor cells of mice studied with Chit-nanoEGCG in comparison to groups studied with epigallocatechin-3-gallate and controls, improved Bax protein expression with a simultaneous reduction in the expression of Bcl-2 protein, and activated caspases [145,146]. According to research, high-efficacy near-infrared (NIR) photothermal treatment has been used on green tea-reduced graphene oxide (GT-rGO) sheets to cure the HT29 and SW48 colon cancerous cells. The polyphenol elements of GT-rGO function as efficient ligands for cargo binding to the surface of cancerous cells, according to cell granularity studies utilizing scanning electron microscopy and flow cytometry. More significant photothermal apoptosis is seen in giant metastatic cancerous cells (SW48) in comparison to smaller metastatic cancerous cells (SW48) by about 20%. Compared to comparable nanoparticles made of carbon, the GT-photo-destruction rGO’s efficiency factor is at least two orders of magnitude higher [147,148]. As a result, tea polyphenols predominantly change molecular pathways during the onset of illness to suppress colon carcinogenesis.

### 6.4. Berberine

Both in vivo and in vitro research on Berberine (BBR) has found that it inhibits the proliferation of cells by causing apoptosis and regulation of the cell cycle along with autophagy. BBR also abolishes cell invasion and metastasis by hindering epithelial–mesenchymal transition (EMT) and down-regulating the protein expression and signalling pathways involved in metastasis [149]. Research has revealed that berberine suppresses cancer cell proliferation by lowering fatty acid synthesis and decreasing biogenesis and extracellular vesicle release. This indicates that berberine might be a viable option for creating novel cancer medications [150]. Another study revealed the berberine-associated regulatory signalling pathways in colon cancer. The researchers hypothesized that ERAL1 and several mitochondrial ribosomal proteins might be effective therapeutic targets for treating this illness [151]. Another study not only identified retinoid X receptor (RXR) as a protein target for berberine, but it also revealed their mode of binding and confirmed that berberine inhibits catenin signalling and cell growth in colon cancer by binding RXR, offering new methods for the development of RXR-based anticancer drugs [152]. Although BBR has positive benefits that may assist in treating malignancies, its effectiveness has not yet been evaluated. Therefore, creating formulations that improve BBR absorption in the intestines might significantly influence cancer treatment.

One of the most popular types of planned cell death that berberine may cause in CRC cells through several routes is apoptosis, which is triggered by caspases [153]. One of these processes is the activation of caspases 3 and 8, which causes the cleavage of poly ADP ribose polymerase (PARP) [154]. Reactive oxygen species (ROS) are produced due to the apoptosis that berberine induces. Additionally, the JNK activation and p38 signalling modulators increase phospho-c Jun, FasL, and t-BID cellular levels during berberine-induced apoptosis. In addition, it promotes caspase 3 cleavage, increases p53 phosphorylation, and inhibits NF-κB activity in vitro [155,156]. The high DNA-binding ability of berberine, which resulted in epigenetic alterations, was thought to be a potential cause of its antitumor impact. Berberine’s potential to fight cancer has been well investigated in various cancer cells. A study discovered that by giving berberine orally to persons with familial adenomatous polyposis, polypectomy subsequently decreased the quantity and size of polyps and interrupted the recurrence of colorectal polyps. Similar studies have shown that berberine suppresses de novo lipogenesis and catenin signalling, which prevent cell development. It binds RXR via a new binding path that improves RXR interaction with nuclear catenin. According to research, nanocarriers improve the pharmacological effects of Brb. Oral administration and sustained/controlled Brb release were increased using nanoparticles made of chitosan, alginate, dextran, and PLGA [155]. Brb-containing solid lipid nanoparticles (SLNs), micelles, nanostructured lipid carriers (NLCs), and liposomes show a significant anti-cancer effect. Brb combined with dendrimer, graphene, gold, and silver nanoparticles have shown potential against cancer cells [155,156]. Thermal treatment and bioimaging are both possible with brb-based carbon dots. BBR may also interfere with the SCAP/SREBP-1 signalling pathway, which is linked to lipid formation in CRC cells [157]. All of the studies above point to a possible mechanism for berberine’s anti-cancer action and its potential therapeutic use in the treatment of colon cancer.

### 6.5. Plumbagin

The medicinal herb Plumbago zeylinica contains a naturally occurring substance called plumbagin that has been used carefully for millennia in Indian Ayurvedic to treat a variety of diseases [158]. Plumbagin has generated much scientific attention owing to its distinguished pharmacological properties as an antibacterial, hypolipidemic, anti-atherosclerotic, leishmanicidal, and anti-cancer agent [159]. It has been regarded as a potential therapy for colon cancer due to its low toxicity and anti-survival concrete action on the colon cancer cell. Plumbagin has been discovered to be a strong radio sensitizer. According to a study, plumbagin strongly impacted HCT116 colon cancer cells, whether it was administered to attach or detached cultures. A different study discovered that plumbagin regulates p65 (NF-κB), which lowers the phosphorylation of Akt, EGFR, and GSK-3β, and causes cell death. Numerous studies have suggested that plumbagin may be a valuable medication for the treatment of colon cancer [160,161].

Thalidomide and bortezomib’s apoptotic effects can be markedly boosted by plumbagin. The chemosensitivity of STAT3-overexpressing malignancies has been theorized to be caused by plumbagin’s inhibition of the STAT3 activation pathway through SHP-1 induction. It has been demonstrated that plumbagin downregulates the expression of NF-κB regulated genes, which causes gastric cancer cells to undergo apoptosis and enhances their susceptibility to the chemotherapy drugs TNF-α and cisplatin [162]. Similar to this, it was shown through an in vitro investigation of HCT15 and HT29 cells that plumbagin can induce apoptosis in colon cancerous cells through triggering of caspase-3 and release of cytochrome c by TNF-α expression and TNF-α facilitate cascade. Plumbagin has also been found to inhibit Akt, EGFR, and GSK-3β activation, which tips the scales in favor of apoptosis by lowering cell viability [163]. Both the stimulation of arrest of the cell cycle in the G1 phase, which was assisted by increases in p53 and p21WAF1/CIP1, and the creation of ROS that triggered apoptosis through the mitochondrial cell death path, had an impact on HCT116 cells [161]. Furthermore, several studies have shown that the expression of several Wnt coactivators and downstream targets is reduced in SW620 cells, indicating that plumbagin inhibits CRC cells by modifying the Wnt signalling pathway [164].

### 6.6. Saikosaponin

Saikosaponin is one of the principal triterpenoid saponins isolated from *Bupleurum falcatum* L. and it displays several pharmacological effects, including immunoregulating, anti-inflammatory, antiviral effects, antibacterial, and anticancer. This natural plant has been utilized to treat fever, the common cold, and hepatitis as part of traditional medicine around the globe. Saikosaponin may show antitumor action by inhibiting cell growth or inducing apoptosis in human breast cancer, colon, and hepatoma cells [165].

Saikosaponin-a (SSa)-induced apoptosis is often restricted to specific cell types, including colon cancer cells. Human colon cancer (HCC) cells, in vitro models, and xenograft animal models have mainly been employed to analyze the antitumor effects of SSa [166]. Additionally, SSa may activate many endoplasmic reticulum (ER) stress receptors. Previous research has indicated that caspase-4 is an upstream control of SSa-induced DNA damage and caspase 4 activations in HCC cells, as well as the concurrent activation of caspase-2 and caspase-8, which is a crucial phase in SSa-triggered apoptosis and leads to caspase-mediated apoptosis in HCC cells, suggesting that it may be a promising CRC therapeutic agent [167]. Like SSa, Saikosaponin-d (SSd) might trigger apoptosis by modifying the expression of apoptotic genes in CRC cells. In addition to laying the framework for understanding the processes behind the SSd-induced apoptosis of CRC cells, the findings may potentially give potential therapy options for colon cancer. SSd therapy alleviates DSS-induced intestinal inflammation by suppressing NF-κB activation and controlling the gut microbiota; furthermore, it dramatically reduces disease activity index (DAI) by expanding colon length and enhancing pathological features and several other metrics [167,168].

## 7. Challenges and Advantages of Nanoformulated Drugs

It has been shown that the matrix barrier, tumor niches such as collagen, fibrosis, tumor heterogeneity along with alternative matrices with varied vasculature, and the weakly vascularized tumor core are impediments to the targeted administration of anti-cancer drugs to solid tumors carrying NPs. The current emphasis of NP-based tumor medicine delivery research is the “active” rather than “passive” targeting of NPs through the enhanced permeability and retention mechanism. Formulations for non-pharmaceutical purposes encounter more distinct issues than pharmaceutical formulations, making their development more challenging. Nanopharmaceuticals are extraordinarily complex chemicals that need careful consideration of carriers, form, pharmacokinetic factors, and inorganic material optimization to meet therapeutic objectives and assure optimal storage. Production on a wide scale of nanotherapeutics demands storage, efficient equipment, and space. However, the more the complexity of the non-pharmaceutical, the greater the manufacturing and acquisition costs. Consequently, consideration must be given to developing nanopharmaceuticals and nano-nutrients cost-effectively. A well-formulated and well-manufactured system is essential to justify the expense of production [51,52,53,169].

**Figure 5:**
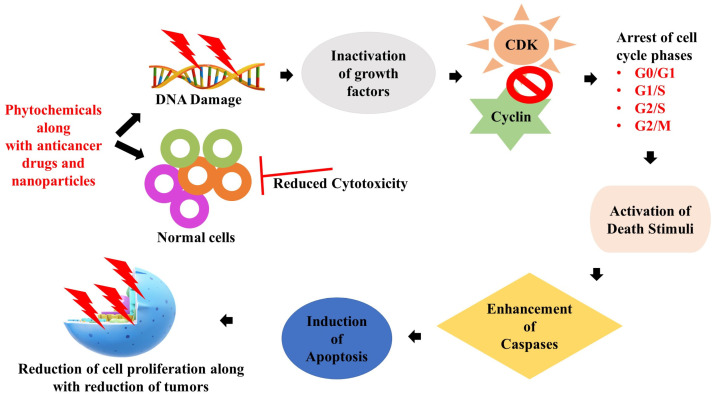
Figure representing the role of phytochemicals in inducing apoptosis via modulation of cell cycle signalling checkpoints.

There are several restrictions associated with CRC treatment. Frequent emphasis is placed on the lack of medication targeting and cell specificity, resulting in unwanted side effects. Nanotechnology might spare patients from a great deal of pain and suffering, battle multidrug resistance, and enhance the killing of tumors. Several experiments are now being conducted to see if nanoformulations might improve the sensitivity and specificity of CRC, as shown in Figure 5. Although monoclonal antibodies (mAb) are often utilized to treat CRC, the utilization of nanoformulated treatments requires more preclinical and clinical investigation. Peptides are also a viable targeting option based on the size and ability of nanoparticle attachment. Although, the use of peptides for CRC, such as the tumor necrosis factor-associated apoptosis-inducing and the targeted peptide RPMrel (CPIEDRPMC) [163,169], has not yet been exhaustively studied. In a current phase 1 investigation, the use of ultra-small silica particles for imaging human brain tumors is being examined. Understanding the principles of silica nanoparticle distribution and excretion in humans might contribute to creating future targeted CRC drugs. Carbon nanoparticles are being investigated as lymph node tracers for CRC to see whether they may increase lymph node yield after surgery [133]. The magnetic resonance of Ferumoxytol-iron oxide nanoparticles is being evaluated as a potential approach for comprehending the spread of cancer [170,171]. With the available information, we can infer that nanotechnology thrives in oncology as clinical research continues to help detect and treat cancer.

Manufacturing may not be a problem for all approved passive targeting products. Still, it may be for active nano-targeting, in which the targeting component must be chemically conjugated to the nanoplatforms and then loaded with drugs for delivery into the tumor and its niche; however, these disputes may be resolved. In addition, the physicochemical characteristics of NPs contribute to toxicity in the human body. The FDA’s regulation of nanomedicines and nanonutraceuticals is a further concern that must be addressed. Currently, the FDA studies nanomedicines in the same manner as it investigates products that do not include nanomaterials. The FDA’s regulatory structure looks insufficient because of the increasing complexity and variety of nanoformulations. There may be concerns about safety, effectiveness, and accurate labeling. Irrespective of the limitations, nano-based delivery of established or novel anti-cancer medicines offers significant promise for improving the treatment of a number of diseases including cancer. Delivery of nanoparticle drugs increases therapeutic effectiveness, hydrophobic drug solubility, and the half-lives of proteins along with unstable chemicals. It also permits regulated and targeted medication release at the tumor site and its niche [169,170,171]. Lastly, it is believed that the aggregation of numerous technologies and collaboration with various experimental and theoretical researchers from the pharmaceutical and academic sectors will expedite the transition of these breakthroughs from the laboratory to the bedside and, ultimately, the marketplace as shown in Table 1 and Table 2. As most anti-cancer drugs are cytotoxic and have a limited therapeutic index, achieving more remarkable survival and quality of life via effective and safe cancer therapy remains a significant problem.

## 8. Conclusions and Future Perspective

Researchers in the last two decades have explored the vast proof that phytochemicals have diverse anticancer effects. Although, its therapeutic use is restricted because of its weak water solubility and low body retention. By adjusting the pharmacokinetics and pharmacodynamics of phytochemicals, phytonanomedicine based on phytochemicals can get around these problems and increase the effectiveness of treatments. By adopting an intelligent, practical, and logical perspective, phytonanomedicine can have a more significant positive therapeutic impact. Using phytochemicals in conjunction with nanotechnology enhances the therapeutic impact and offers a novel approach to solving the challenging economic and environmental issues associated with nanotechnology. Consequently, a promising strategy is to combine phytochemicals with nanotechnology. However, there are still issues with nanotechnology that need to be resolved.

Even though nanomedicine has advanced to new heights, pharmacological as well as kinetic investigations on tumors are difficult due to heterogenicity and complicated tumor anatomy. Furthermore, it is challenging to create tumor-imitating 3D spheroid models that are typically implicated in tumor metastasis and the development of resistance to drugs within the tissue. This makes in vitro analysis on the treatment of cancer extremely challenging. Theranostic approaches are increasingly in demand because they provide real-time imaging besides therapy, allowing for additional individualized investigation of phytomedicine. Additionally, as this will greatly increase the formulation’s safety, efficacy, and stability, the regulatory features of phytochemical treatment may continue to be important in combination with nanotechnology. Combining current methods will be a key component of anticancer therapy in the future. It is crucial to identify which techniques combine well in order to obtain the highest anticancer effect. The most effective combination therapy to treat cancer can be created by knowing the precise mechanisms by which therapeutics eliminate tumors. Currently, NP immunotherapy is the most effective anticancer method. It is expected that the field of nanotechnology can be used to improve the anticancer effectiveness of traditional immunotherapy. PEGylation of nanomedicine can be helpful since it allows the nanoparticles to bypass immunological macrophages and lengthens the half-life of phytochemicals in the blood. According to recent studies, cancer patients who receive cancer immunotherapies experience some pathophysiological limitations. In this situation, developing phytonanomedicines with immunomodulatory properties, combining phytonanomedicine with immunotherapy, or administering nanoformulations for CART treatment within the body of the patient might assist to enhance the outcomes of immuno-oncological interventions along with boosting the proportion of long-term survivors. According to numerous studies, conventional medicines can effectively target rapidly reproducing cancer cells while sparing dormant CSCs in tumor masses. Research indicates that phytochemicals have potent anti-CSC effects in this situation. Therefore, targeting both cancer cells and CSCs separately with a mix of conventional chemotherapeutics and phytochemicals may be a successful strategy for total tumor remission. Furthermore, simultaneous modulation of CSCs and cancerous cells as well as their microenvironment can be achieved through the phytochemicals co-delivery with siRNA or miRNA using nanoparticles, which may improve the therapeutic index. Additionally, a phytochemical can be nanoformulated with a *photoluminescent* compound and then coupled to a peptide or antibody to provide diagnosis and targeted treatment at the same time. Therefore, by decreasing side effects, the development of such a novel combinational nanoformulation can aid in improving patient compliance.

Designing a unique phytochemical-nanocarrier that enhances medication release or absorption in cancerous cells is the ultimate goal for nanomedicine delivery in order to obtain a stronger treatment benefit as well as fewer adverse consequences in clinical situations. The nanoformulation can be coupled to some targeting ligands to produce active targeting, enhancing phytonanomedicine’s potential to target tumors. Additionally, the potential for site-specific medication delivery via nanomedicine prevents nonspecific drug administration that helps to control toxicity. Therefore, research into phytonanomedicine development, which may particularly release medication to the tumor site may become more active. Numerous studies are currently focusing on various kinds of nanocarriers, loaded with phytochemicals for efficient tumor targeting; however, the main issues for clinical translation are safety, cost, chronic toxicity, and large-scale production. To develop the next generation of cancer therapeutics, more study is therefore needed to comprehend and enhance the design, production, and physiological relations of phytonanomedicine. Additionally, it is necessary to build smart tactics to spread phytonanomedicine to as many patients as possible through the combined efforts of consortiums made up of academics/researchers, physicians, pharmaceutical corporations, and regulatory agencies. To improve the effectiveness and outcomes of phytonanomedicine as an anticancer medication, this publication seeks to streamline the function of nanomedicine in translational cancer research.

## Figures and Tables

**Table 1:** Table representing some of the phytochemicals used for preclinical analysis for CRC [31].

Sl No.	Name of Phytochemical	Chemical Formula	Source	Cancer Type
1.	Curcumin	C_21_H_20_O_6_	*Curcuma longa*	Colorectal
2.	Quercetin	C_15_H_10_O_7_	Olive oil, citrus fruits, apples, and onions.	Colon
3.	Genistein	C_15_H_10_O_5_	Tofu, fava beans, soybeans, kudzu.	Colon
4.	β-carotene	C_40_H_56_	Carrots, Sweet potatoes, winter squash	Colon
5.	Lycopene	C_40_H_56_	Tomatoes, watermelon	Colon
6.	Ursolic acid	C_30_H_48_O_3_	Apple peel, cranberry juice, grape skin, basil, rosemary, thyme, and oregano.	Colorectal
7.	Piperine	C_17_H_19_NO_3_	Fruits and roots of *Piper nigrum* L. and *Piper longum* L.	Colon
8.	Piperlongumine	C_17_H_19_NO_5_	Fruits and roots of the long pepper plant	Colon
9.	Thymoquinone	C_10_H_12_O_2_	Seeds of *Nigella sativa* L.	Colon
10.	Sulforaphane	C_6_H_11_NOS_2_	Cruciferous vegetables	Colon

**Table 2:** Table representing a few of the applications of nanoformulated phytochemicals in colon cancer [172].

Sl. No.	Name of Phytochemical	Method of Delivery
1.	Curcumin	Liposomal formulations, hollow capsules, polyvinylpyrrolidone-conjugate micelles
2.	Epigallocapechin Gallate	Gold nanoparticles, lipid nanoparticles, polyethylene glycol
3.	Ellagic acid	Polyethylene glycol, poly lactic-co-glycolic acid nanoparticle
4.	Β-Lapachone	Polyethylene glycol-polyactide micelles
5.	Eugenol	Magnetic nanoparticle, Nanoemulsion

## Data Availability

Not applicable.
